# Predicting cognitive change using functional, structural, and neuropsychological predictors

**DOI:** 10.1093/braincomms/fcaf155

**Published:** 2025-04-18

**Authors:** Laurie Décarie-Labbé, Samira Mellah, Isaora Z Dialahy, the CIMA-Q Consortium, Pierre Bellec, Sylvie Belleville, Christian Bocti, Frédéric Calon, Howard Chertkow, Louis Collins, Stephen Cunnane, Simon Duchesne, Pierrette Gaudreau, Serge Gauthier, Sébastien S Hébert, Carol Hudon, Marie-Jeanne Kergoat, Andréa C LeBlanc, Nicole Leclerc, Naguib Mechawar, Natalie Philips, Jean-Paul Soucy, Thien T D Vu, Louis Verret, Juan M Villalpando, Sylvie Belleville

**Affiliations:** Research Center, Institut universitaire de gériatrie de Montréal, Montreal, Quebec, Canada, H3W 1W5; Department of Psychology, Université de Montréal, Montreal, Quebec, Canada, H3C 3J7; Research Center, Institut universitaire de gériatrie de Montréal, Montreal, Quebec, Canada, H3W 1W5; Research Center, Institut universitaire de gériatrie de Montréal, Montreal, Quebec, Canada, H3W 1W5; Research Center, Institut universitaire de gériatrie de Montréal, Montreal, Quebec, Canada, H3W 1W5; Department of Psychology, Université de Montréal, Montreal, Quebec, Canada, H3C 3J7

**Keywords:** functional magnetic resonance imaging, brain structure, neuropsychological measures, mild cognitive impairment, subjective cognitive decline

## Abstract

To effectively address Alzheimer’s disease, it is crucial to understand its earliest manifestations, underlying mechanisms and early markers of progression. Recent findings of very early brain activation anomalies highlight their potential for early disease characterization and predicting future cognitive decline. Our objective was to evaluate the value of brain activation—both individually and in combination with structural and neuropsychological measures—for predicting cognitive change. The study included 105 individuals from the Consortium for the Early Identification of Alzheimer’s Disease–Quebec cohort who exhibited subjective cognitive decline or mild cognitive impairment. Cognitive decline was assessed by calculating the slope of Montreal Cognitive Assessment scores using regression models across successive assessments, and individuals were characterized as either decliners or stable based on clinically reliable change. We evaluated cognitive decline predictions using unimodal models for each class of predictors and multimodal models that combined these predictors. Functional activation emerged as a strong predictor of cognitive change (R²=52.5%), with 87.6% accuracy and 98.7% specificity, performing comparably to structural and neuropsychological measures. Although the unimodal functional model exhibited high specificity, indicating that functional abnormalities frequently predict future decline, it had low sensitivity (60%), meaning that the absence of abnormalities does not rule out future decline. Multimodal models provided greater explanatory power than unimodal models and greater sensitivity than the functional model. These findings highlight the potential role of early brain activation anomalies in the early detection of future cognitive changes, offering valuable insights for clinicians and researchers in assessing cognitive decline risk and refining clinical trial criteria.

## Teaser

Décarie-Labbé *et al*. suggest hyperactivation could serve as an early marker of Alzheimer’s disease. Their multimodal study identified brain activation as a strong predictor of cognitive decline in at-risk individuals, with excellent specificity but lower sensitivity. Predictive power and sensitivity increased when activation was combined with structural and neuropsychological measures.

## Graphical Abstract

**Graphical Abstract:**
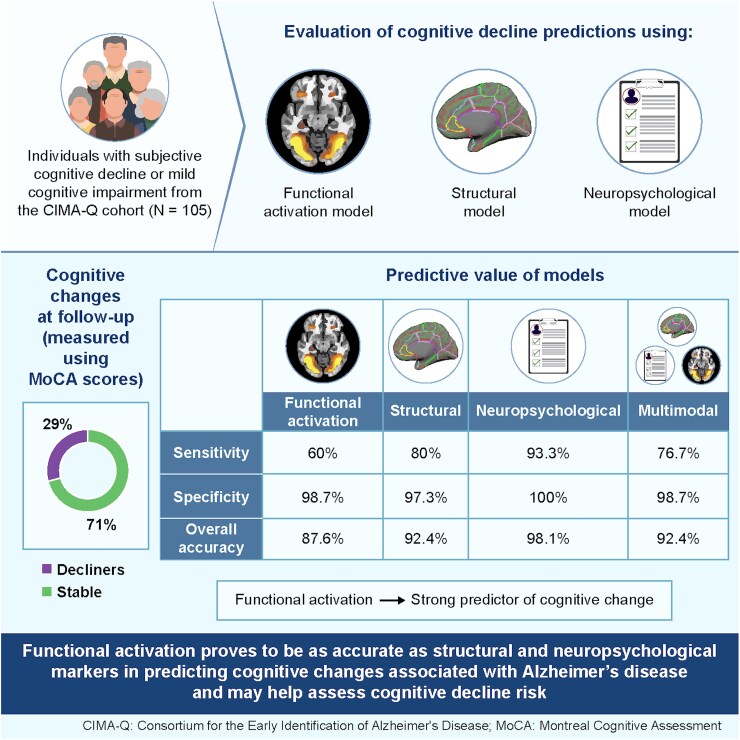

## Introduction

Alzheimer's disease is characterized by a prolonged prodromal phase that often begins several years before clinical diagnostic criteria are met.^1^ To effectively address Alzheimer's disease, it is essential to understand the earliest manifestations during this prodromal stage, as they can provide valuable markers for predicting disease progression. Subjective cognitive decline (SCD) and mild cognitive impairment (MCI) have gained significant attention in this context. Individuals with MCI report memory issues and exhibit cognitive impairments on clinical measures. However, these impairments are not severe enough to meet criteria for dementia, as they do not significantly interfere with the individual’s daily functioning. In contrast, dementia involves significant cognitive decline that interferes with independent living and daily activities. Individuals with SCD, considered a precursor to MCI, experience subjective complaints without objective evidence of impairment. Given many individuals with SCD or MCI eventually experience cognitive decline, potentially indicating underlying Alzheimer's disease,^2-5^ these conditions are valuable for studying the early stages of the disease and identifying markers of future decline.

The presence of early anomalies in brain activation suggests a potential role in characterizing the initial stages of Alzheimer's disease and detecting future cognitive decline.^6^ Recent studies indicate that individuals with MCI exhibit higher levels of brain activity during task-related activation protocols compared with cognitively healthy controls (HC). This phenomenon, known as hyperactivation,^7-11^ is most commonly observed during memory encoding and retrieval tasks, although it has also been reported in tasks assessing executive functions. Notably, hyperactivation has also been observed in individuals with SCD,^7,10-12^ suggesting it might represent one of the earliest manifestations of Alzheimer's disease–related brain changes. This hyperactivation is thought to reflect either early excitotoxicity,^13-17^ an early neural compensation^10,18-21^ or a combination of both.^22-24^

While many individuals with SCD or MCI experience cognitive decline,^2-5^ some remain stable or even revert to normal cognitive function. In these non-progressors, cognitive symptoms may stem from various other causes, such as functional cognitive disorder. This condition, characterized by stable or fluctuating but non-progressive cognitive symptoms, is increasingly recognized as a common issue in clinical practice^25^ and is distinct from neurodegenerative processes. One crucial question is whether hyperactivation can effectively differentiate between those who will eventually decline and those who will not. This raises the additional question of whether hyperactivation can predict individual progression on its own or when combined with other markers. Another key consideration is whether brain activation serves as a more specific marker, effectively identifying those who will remain stable (higher specificity), or as a more sensitive marker, better at identifying those who will decline (higher sensitivity), thus minimizing false negatives.

Numerous studies, including meta-analyses and reviews,^26-30^ have sought to identify measures that can predict progression to dementia. These studies employ longitudinal assessments of individuals to evaluate predictors of progression to dementia.^31-37^ Key predictors of future cognitive decline include amyloid or tau levels,^38-41^ volumetric measures of brain regions, such as the entorhinal cortex, hippocampus, anterior and posterior cingulate and frontal and temporal lobes,^42,43^ as measured by structural MRI, as well as neuropsychological performance.^44-47^ These markers typically demonstrate a strong capacity to predict progression from MCI to dementia.^26-30^ However, these markers of progression are typically assessed only in individuals with MCI, leaving a gap in understanding their applicability to those with SCD.

Given that hyperactivation is observed early in the disease process, even at the SCD stage, it holds promise as an early indicator of future cognitive decline. To our knowledge, no studies have specifically evaluated the predictive value of activation parameters for identifying individuals with SCD or MCI who will experience subsequent cognitive decline. Additionally, it remains unclear how these activation parameters compare to more widely used markers, such as neuropsychological measures and structural brain changes. Another crucial question is whether combining functional activation with these other markers improves prediction accuracy. Since task-related brain activation reflects the brain processes engaged during task performance, it could potentially complement other measures. Several studies have demonstrated that multimodal prediction models outperform unimodal models, supporting the notion that Alzheimer's disease is multifactorial.^36,48-52^ This suggests that incorporating diverse markers is optimal, as it may better capture the various underlying pathophysiological mechanisms at play. If hyperactivation represents a unique mechanism, it may offer additional predictive value when used alongside structural and neuropsychological markers.

This study aimed to investigate the value of functional activation in predicting cognitive decline among individuals with SCD or MCI. Additionally, the study sought to assess and compare the predictive accuracy of MRI-based structural indicators and neuropsychological measures, which are non-invasive and relatively accessible. The study measured predictive accuracy, sensitivity and specificity for each model. Additionally, it assessed the added value of multimodal models that combine activation, structural and/or neuropsychological measures, compared with unimodal models. Cognitive decline was assessed using the Montreal Cognitive Assessment (MoCA), employing both continuous and binary approaches. A regression model was applied to participants’ longitudinal MoCA scores across all available timepoints to calculate the corresponding slope. Participants were also classified as decliners or stable using a reliable change index, indicating a clinically meaningful difference.^53^ We hypothesized that activation parameters would predict cognitive change (i.e. explained variance and decliner status) and that combining markers would improve the prediction of cognitive decline over unimodal models.

## Materials and methods

### Source of data

This article follows the guidelines outlined in the Transparent Reporting of a multivariable prediction model for Individual Prognosis or Diagnosis (TRIPOD).^54^ Data were collected from September 2016 to September 2022 through the Consortium for the Early Identification of Alzheimer’s Disease (CIMA-Q), which provides access to its data upon request (http://www.cima-q.ca/en/home/).^55^ CIMA-Q is an ongoing multisite longitudinal observational cohort study with over 380 participants, including cognitively healthy individuals as well as those with SCD, MCI or probable Alzheimer's disease. The main objective of CIMA-Q is to understand and characterize the first stages of Alzheimer's disease, contribute to its early diagnosis, and identify new therapeutic targets.

### Participants

Participants in the CIMA-Q study were recruited from the community through various means, including electronic media and memory clinics in the province of Quebec, Canada. Their consent was obtained in accordance with the Declaration of Helsinki and was approved by the Comité d’éthique de la recherche vieillissement-neuroimagerie. Inclusion criteria varied based on the participants’ cognitive status: Participants with MCI met the criteria of the National Institute on Aging and Alzheimer’s Association (NIA-AA) working group for MCI,^56^ which included the following: (i) self-reported memory complaint; (ii) objective memory impairment on the delayed portion of the Logical Memory subtest of the Wechsler Memory Scale,^57^ using an education-adjusted threshold (≤ 2 for 0–7 years of education, ≤ 4 for 8–15 years and ≤ 8 for 16 or more years); (iii) a MoCA^58^ score between 20 and 25; (iv) a Clinical Dementia Rating Scale of 0.5. Criteria for participants with SCD were: (i) self-reported memory complaint and concern; (ii) a normal education-adjusted threshold on the delayed portion of the Logical Memory subtest (≥ 3 for 0–7 years of education, ≥ 5 for 8–15 years, and ≥ 9 for 16 or more years); (iii) a MoCA score > 26 and (iv) a Clinical Dementia Rating Scale score of 0.

All participants were French or English speakers, right-handed and free from dementia or other conditions that could affect the brain. Additionally, they had no contraindications for undergoing MRI procedures. For further details on the inclusion and exclusion criteria used by CIMA-Q, refer to the study by Belleville *et al*.^55^ For this study, we selected data from participants who met the criteria for MCI or SCD, had completed at least one fMRI assessment, and had undergone at least one two-year follow-up assessment.

### Outcome variables

Cognitive change was measured using the MoCA, a widely recognized tool in both research and clinical settings,^59^ validated for the Quebec population.^58^ The MoCA is known for its sensitivity to change,^53^ making it a valuable indicator of cognitive decline. Participants underwent MoCA assessments at the time of their initial scan and during subsequent visits, which were scheduled every 2 years thereafter.

To assess cognitive change, we used two metrics: First, we calculated cognitive change scores by fitting a regression model to participants’ MoCA scores across all available time points, with the resulting slope representing cognitive change on a continuous scale. Second, as a binary approach, we applied the findings from the study by Krishnan *et al.*,^53^ which identified a reliable change of 1.73 points over 3.5 years as a clinically meaningful difference. This value was prorated based on each participant’s follow-up duration, allowing us to classify participants as either experiencing a clinically significant cognitive decline (referred to as ‘decliners’) or maintaining stable cognition (referred to as ‘stable’).

### Neuroimaging predictors

#### Data acquisition and image processing

Brain imaging was conducted using three Tesla magnetic field scanners (Siemens Healthcare TrioTim or Philips Medical Systems Achieva and Ingenia). The acquisition and harmonization of structural sequences across scanning sites followed the Canadian Dementia Imaging Protocol (www.cdip-pcid.ca).^60^ Brain activation was measured during memory encoding.^7^ Detailed descriptions of image acquisition parameters and processing are available in Belleville *et al*,^61^ and in Corriveau *et al*l^61-63^ for functional images, and in Caillaud *et al*^7^ and Corriveau *et al*^63^ for structural images. A full list of sequences is provided in the Supplementary Materials.

T1-weighted images were captured in the sagittal plane, with an echo time (TE) of 3.3 ms for Philips and 2.98 ms for Siemens scanners. The repetition time (TR) was set to 2300 ms, with a rotation angle of 9 degrees. The voxel size was 1 × 1 × 1 mm, and the matrix and field of view were 256 × 248 mm for Philips and 256 × 256 mm for Siemens systems. Task-related fMRI images were obtained in the anteroposterior commissure orientation (AC-PC) at -20 degrees, using echo planar imaging (EPI) sequences sensitive to the Blood Oxygenation Level Dependent contrast. The interslice gap was 0.3 mm (Philips) or 0 mm (Siemens). The TE was 25 ms, the TR was 2500 ms and the rotation angle was 90 degrees. The voxel size was 3 × 3 × 3 mm, with a field of view of 240 × 240 mm (Philips) or 222 × 222 mm (Siemens) and a matrix size of 80 × 80 mm (Philips) or 74 × 74 mm (Siemens).

#### Functional activation predictors

The fMRI task involved an associative memory encoding task during the scan, which lasted approximately 10 min. During encoding, 78 images of common objects were sequentially displayed on a screen, each positioned in one of four grid locations for 3 s. The inter-stimulus intervals varied between 0.5 and 18.5 s. Participants were instructed to remember both the object and its position. To serve as controls, 39 gray squares were intermittently displayed. The sequence and timing of stimuli and controls were optimized using Optseq2. Following the encoding phase, participants moved to an adjacent testing room for a retrieval task, conducted about 10 min after the encoding session. During retrieval, participants were shown the 78 previously studied items alongside 39 new ones, all displayed in the centre of the screen. For each item, participants had to indicate whether it was presented during the learning phase using a yes-no response key and identify its original position in the grid using a four-button keypad.

Functional data were analysed using Statistical Parametric Mapping version 12 (SPM12) software within MATLAB 9.4. First, data were realigned to the initial image captured during the session, and an average image was generated. The realigned volumes were then co-registered with participants’ structural T1 images, normalized to the Montreal Neurological Institute stereotactic space with a voxel size of 3 mm^3^ and spatially smoothed using an 8 mm full-width Gaussian filter at half maximum. To remove low-frequency signal deviations, a high-pass filter with a 128 s period was applied. Participants whose fMRI activation images failed quality control—due to a total displacement higher than 3 mm and/or framewise displacement exceeding 1 mm—were excluded from the analyses. Head displacement did not differ significantly between individuals with SCD and MCI or between decliners and non-decliners. As a result, it was excluded from the models.

The associative memory contrast was calculated by subtracting the activation associated with control items from the activation associated with items that were both well-recognized and correctly positioned. This contrast was subjected to a family-wise correction, with a significance threshold set to *P* < 0.05 for clusters.

The functional variables included as predictors in the models were derived from regions of interest (ROIs) identified in our previous study as showing hyperactivation or hypoactivation relative to HC.^7,64^ These ROIs included the left hippocampus, superior parietal lobe, inferior temporal lobe, right superior temporal pole, left inferior frontal gyrus, superior frontal lobe and middle temporal lobe. ROI masks were created using the PickAtlas toolbox.^65^

#### Structural brain predictors

Cortical reconstruction and volumetric segmentations were conducted using the FreeSurfer 6.0 package. Normative morphological data were obtained by comparing the study participants’ data to large-scale datasets of HC aged 18–94 from the *Alzheimer’s Disease Neuroimaging Initiative* (ADNI) and the *Australian Imaging, Biomarkers and Lifestyle study of aging* (AIBL) databases.^66,67^

The structural variables used as predictors in the models were selected based on ROIs identified in McEvoy’s work,^68^ which demonstrated significant atrophy in individuals with amnestic MCI compared with HC. These ROIs were also found to be strong predictors of cognitive decline in individuals with MCI and of progression from MCI to Alzheimer's disease,^69^ as well as from HC to MCI.^70^ The ROIs included cortical thickness of the entorhinal cortex, middle temporal gyrus, bank of superior temporal sulcus, superior temporal gyrus, isthmus cingulate, lateral orbitofrontal gyrus, medial orbitofrontal gyrus and the mean volume of the hippocampus.

### Neuropsychological predictors

The selection of neuropsychological tests included as predictors in the models was based on literature reviews identifying key neuropsychological predictors of cognitive decline in individuals with MCI.^26,61^ Episodic memory was assessed using the sum of Free and Cued Recall scores of the word-list task of the Memoria battery (FCR)^71^ and the delayed recall score from the Rey-Taylor auditory-verbal test.^72,73^ Executive functions were measured with the ratio of time to complete Trail A and Trail B of the Trail Making Test^74^ and the Alpha-Span Task.^75^ Semantic memory was measured with the animal fluency test of the Delis-Kaplan Executive Function System (D-KEFS)^76^ and correct naming score from the Boston Naming Test.^77^

### Sample size

A total of 112 participants met the study criteria. Of these, seven were excluded from the analysis because their fMRI activation images failed quality control or exhibited a total displacement exceeding 3 mm and/or a framewise displacement higher than 1 mm. The final sample for analysis comprised 105 participants, including 32 individuals with MCI and 73 with SCD.

### Statistical analysis

All statistical analyses were conducted using the R software package (http://www.R-project.org). The analysis code used in this study is provided in the Supplementary materials.

As preliminary analyses, T-tests were performed to compare decliners and stable participants at baseline on demographic characteristics and predictive measures. The normality of the distribution of the MoCA scores and predictors was verified using the Kolmogorov–Smirnov test, supported by visual inspection of histograms and evaluation of skewness and kurtosis. When normality assumptions were not met, M-estimation robust regressions were used in forward stepwise regression analyses to minimize the influence of outliers.^78,79^ The correlation matrix of predictors within each modality—functional, structural and neuropsychological—was also examined, and variables that were highly correlated (*r* > 0.8) were excluded. To address missing data, we applied the ClustImpute imputation method, which applies K-means clustering with integrated imputation, providing a robust approach to handling missing values within the dataset.

To analyse unimodal predictive models, we began by conducting three separate forward stepwise regression analyses for functional, structural and neuropsychological variables, each controlling for scanner site and intercept (*P*-to-enter = 0.05). The objective was to identify the predictor(s) for each modality that would be included in the unimodal models. We used statistical significance (*P* < 0.05) as a selection criterion. Following this, separate generalized mixed linear models were applied for unimodal models, incorporating age, education and clinical status as control variables, with sex included as an interaction variable. Sex was included as an interaction term, as it is increasingly studied in Alzheimer's disease research due to notable differences between men and women. Women are more likely to develop Alzheimer's disease and tend to experience faster cognitive decline.^80-82^ Accounting for sex effects ensures more accurate findings and avoids misrepresentation of the underlying factors influencing disease progression in each group. The random effects in these models accounted for both the intercept and the measurement time of the MoCA. Performance indices, including the determination coefficient (R²) and Akaike’s Information Criterion (AIC), were calculated for each model.

We then conducted robust mixed logistic regressions, incorporating clinical status as a control variable and accounting for the assumption of unequal variance among observations. The binary categorical outcome variable was decliner status (i.e. decliner versus stable). To determine the optimal cut off for distinguishing between decliners and stable individuals, we employed the Youden statistic (Youden’s J) for each prediction model. The same predictors used in the continuous analyses were included as fixed effects, while the intercept was treated as the random effect in each model. We estimated global odds ratios, overall classification accuracy, sensitivity, and specificity to assess the models’ predictive capacity in differentiating individuals who experienced clinically significant decline on the MoCA from those who did not, evaluating specificity and sensitivity separately.

For the multimodal models, we first conducted forward stepwise regression analyses to select predictors from functional activation, structural and neuropsychological measures identified in the respective unimodal models. This process aimed to identify the predictors to be included in the multimodal models. Separate generalized mixed linear models were then applied to the multimodal models, controlling for age, education and clinical status, with sex included as an interaction variable. The unimodal and multimodal models were compared using the R^2^ difference test, which evaluates the generalized explained variance between two candidate mixed regressions.^83^ This comparison assessed whether combining modalities provided additional value compared with a single modality. Additionally, robust mixed logistic regressions were conducted, incorporating clinical status as a control variable and predicting declining status (decliners versus stable).

## Results

### Preliminary analyses

Table 1 provides an overview of participant characteristics, follow-up information and decliner status. The mean slope of decline was −0.0054 with a standard deviation of 0.0238 (SCD: M = −0.0047, SD = 0.022; MCI: M = −0.0069, SD = 0.028). According to clinically meaningful criteria, 30 individuals (19 SCD and 11 MCI) were identified as decliners (29%), while 75 (54 SCD and 21 MCI) were classified as stable (71%). There were no significant differences between the decliner and stable groups in terms of education, sex ratio and clinical status ratio. However, the decliner group was significantly older than the stable group (*P* = 0.032). While there were no statistical differences between the groups for functional and neuropsychological measures used as predictors, decliners had a significantly smaller hippocampal volume compared with stable individuals (*P* = 0.002; see Table 2). Table 3 provides a comparison of stable individuals and decliners across other neuropsychological measures.

**Table 1:** Demographic characteristics at baseline

	SCD (*n* = 73)	MCI (*n* = 32)	Group effect (partial eta2)	Stable (*n* = 75)	Decliners (*n* = 30)	Group effect (partial eta^2^)
MoCA: M (SD)	27.52 (1.42)	24.75 (2.16)	**<0.001**	26.76 (1.88)	26.47 (2.60)	ns (0.521)
MoCA slope: M (SD)	−0.005 (0.022)	−0.007 (0.028)	ns (0.671)			
Age in years: M (SD)				72.68 (4.58)	74.96 (5.48)	**0.032**
Education in years: M (SD)				15.63 (3.48)	14.57 (3.37)	ns (0.158)
Sex: female, male				50, 25	18, 12	ns (0.518)
Clinical status at baseline: SCD, MCI				54, 21	19, 11	ns (0.383)

Note: Bolded values indicate statistically significant results (*P* < 0.05); ns, not significant.

**Table 2:** Baseline functional, structural and neuropsychological measures

	Stable (*n* = 75)	Decliners (*n* = 30)	Group effect (partial eta^2^)
Functional model			
Activation in the left hippocampus	0.14 (0.25)	0.17 (0.32)	ns (0.716)
Activation in the left inferior frontal gyrus	0.27 (0.28)	0.21 (0.32)	ns (0.286)
Structural model			
Cortical thickness of the middle temporal gyrus	2.72 (0.10)	2.74 (0.10)	ns (0.445)
Cortical thickness of the medial orbitofrontal gyrus	2.34 (0.11)	2.35 (0.10)	ns (0.756)
Cortical thickness of the entorhinal cortex	3.43 (0.26)	3.38 (0.27)	ns (0.313)
Cortical thickness of the lateral orbitofrontal gyrus	2.53 (0.12)	2.51 (0.12)	ns (0.430)
Mean volume of the hippocampi	3696 (383)	3440 (347)	**0.002**
Neuropsychological model			
Sum of free and cued recall scores	13.32 (1.86)	11.86 (2.67)	ns (0.156)

Bolded values indicate statistically significant results (*P* < 0.05); ns, not significant; Measures means, with standard deviations in parentheses. Cortical thickness is expressed in millimeters (mm), while volume of the hippocampi is expressed in cubic millimeters (mm³).

**Table 3:** Baseline neuropsychological measures per cognitive domain

	Stable (*n* = 75)	Decliners (*n* = 30)	Group effect (partial eta^2^)
Episodic memory			
Sum of free and cued recall scores	13.32 (1.86)	11.86 (2.67)	ns (0.156)
Delayed recall score (RAVLT)	9.68 (3.27)	9.03 (3.37)	ns (0.953)
Executive functions			
Trail Making Test (Trail A/Trail B)	2.15 (0.99)	2.18 (0.61)	ns (0.296)
Alpha-Span Task	59.45 (17.49)	54.97 (20.35)	ns (0.953)
Semantic memory			
Animal fluency test (D-KEFS)	20.91 (6.73)	17.70 (3.62)	ns (0.086)
Boston Naming Test	52.13 (5.56)	50.50 (6.98)	ns (0.891)

ns, not significant; Measures means, with standard deviations in parentheses.

The MoCA scores and most predictors were normally distributed with adequate residual distributions. However, the Animal fluency test, the Boston Naming Test and the Trail-Making Test showed slight deviation from normality, with slightly asymmetric distributions (*P* = 0.01). To address this deviation, the M-estimation approach was used in the forward stepwise regression analyses for neuropsychological predictors. All assumptions of the robust logistic regression approach were satisfied, including linearity of the log-odds relative to the independent variables, absence of severe multicollinearity and independence of errors. The correlation matrix of predictors within each modality showed no correlations exceeding 0.8, thus no variables were excluded based on correlation.

### Predictive models

#### Unimodal predictive models

Table 4 presents the significant predictors, the mixed linear models and the mixed logistic regression results. Although sex was included as an interaction variable in the analysis, it did not have a significant effect and is therefore not discussed further.

**Table 4:** Unimodal predictive models of cognitive change

Models	*F* test	*P*	R2	AIC	Effect size	OR	ACC	SEN	SPE			
							(%)	(%)	(%)	*β*	*T stat*	*P*
Functional model	*F*(13, 252) = 42.32	**<0.001**	0.525	471.8	2.027	111	87.6	60	98.7			
Activation in the left hippocampus										−0.006	−3.31	**0.001**
Activation in the inferior frontal gyrus										0.005	2.52	**0.014**
Structural model	*F*(22, 259) = 38.56	**<0.001**	0.605	552.8	2.941	146	92.4	80	97.3			
Cortical thickness of the middle temporal gyrus										−0.009	−4.18	**<0.001**
Cortical thickness of the medial orbitofrontal gyrus										−0.005	−2.31	**0**.**023**
Cortical thickness of the entorhinal cortex										0.005	2.74	**0.007**
Cortical thickness of the lateral orbitofrontal gyrus										0.007	3.51	**<0.001**
Mean volume of the hippocampi										0.005	3.53	**<0.001**
Neuropsychological model	*F*(10, 274) = 53.93	**<0.001**	0.53	580.7	1.864	1050	98.1	93.3	100			
Sum of free and cued recall scores										0.006	3.38	**0**.**001**

Bolded values indicate statistically significant results (*P* < 0.05); β (beta) represents the regression coefficient, showing the strength and direction of the relationship between the predictor and the response. The t-stat measures the significance of each coefficient, while *P* indicates the probability of observing such extreme test statistics under the null hypothesis that the coefficient is zero. The *F*-test evaluates the overall significance of the model. R2 indicates the proportion of variance explained by the model. AIC (Akaike’s Information Criterion) measures the model fit, with lower values indicating better fit. Effect size is represented by Cohen’s d. ACC, overall accuracy; OR, odds ratio; SEN, sensitivity; SPE, specificity.

**Figure 1:**
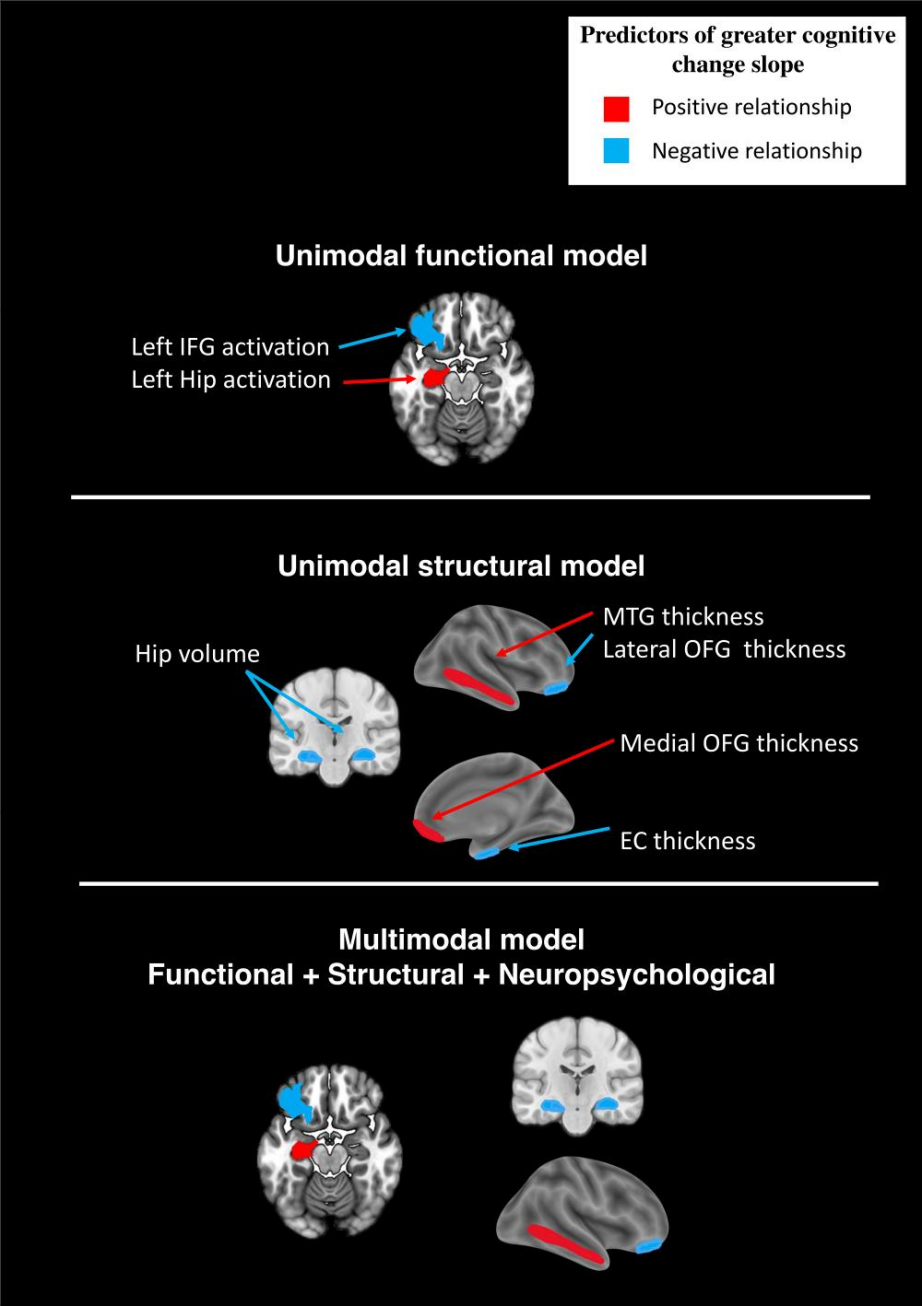
**Regions of interest in functional and structural prediction models.** Recall scores: sum of free and cued recall scores. Thickness: average cortical thickness, calculated across left and right hemispheres, based on cortical regions defined in the DKT labeling protocol. A positive relationship indicates a relationship between cognitive decline and volumetry/activation in the direction of a greater decline associated with a higher volume/activation; a negative relationship indicates a relationship between cognitive decline and volumetry/activation in the direction of a greater decline associated with a lower volume/activation. EC, entorhinal cortex; Hip, hippocampus; IFG, inferior frontal gyrus; lateral OFG, lateral orbitofrontal gyrus; medial OFG, medial orbitofrontal gyrus; MTG, middle temporal gyrus.

**Figure 2:**
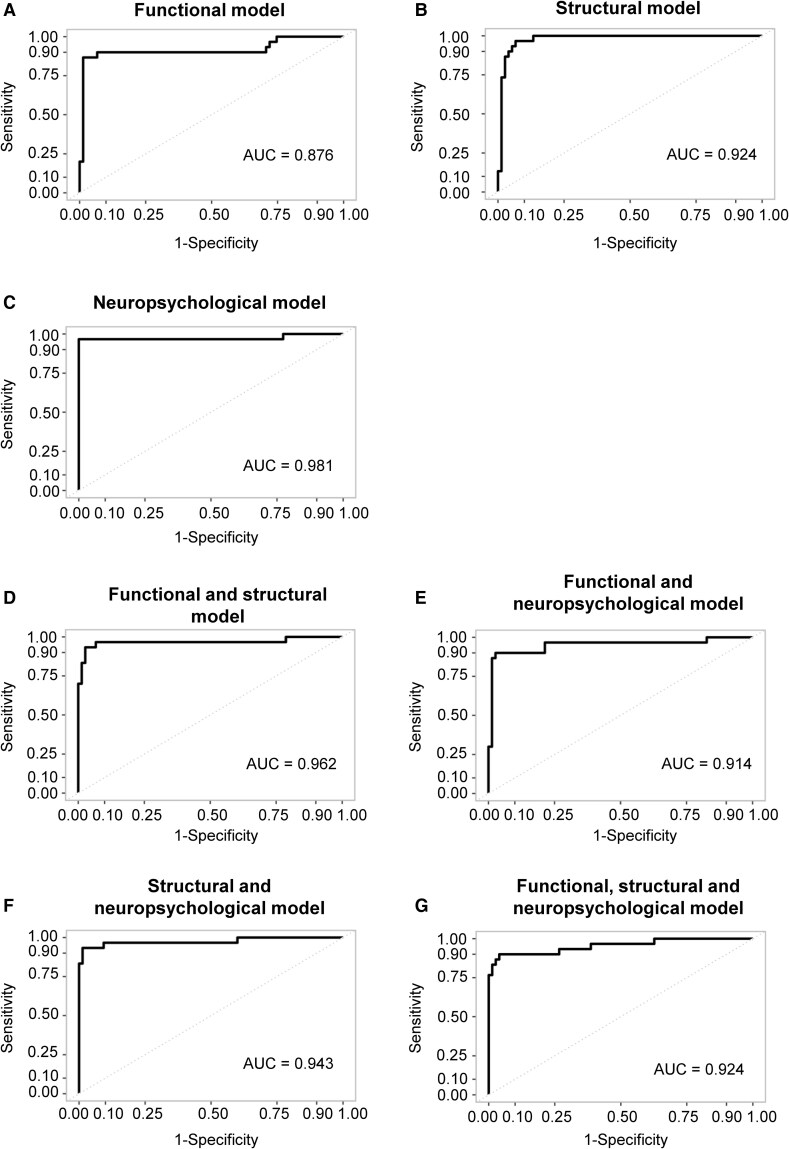
**ROC curves.** The dashed gray line represents the reference line. Unimodal models: (**A**) functional model, (**B**) structural model and (**C**) neuropsychological model. Multimodal models: (**D**) functional and structural model, (**E**) functional and neuropsychological model, (**F**) structural and neuropsychological model and (**G**) functional, structural and neuropsychological model. AUC, area under the curve; ROC, receiver operating characteristic.

In the functional activation stepwise model, two predictors emerged as significant predictors of greater cognitive change slope: higher activation in the left hippocampus and a lower activation in the left inferior frontal gyrus (see Fig. 1 for a visual representation of the two predictors). The unimodal functional model was significant, *F*(13 252) = 42.32, *P* < 0.001, demonstrating a huge effect size (*d*  *=* 2.027)^84^ and explaining 52.5% of the variance in cognitive change. The AIC reflected good quality of fit and moderate complexity (AIC: 471.8). When examining the functional activation model’s ability to differentiate between decliners and stable individuals, the odds ratio for the overall model was 111, *P* < 0.001. With a cut off of 0.587, the model achieved an overall classification accuracy of 87.6%, specificity of 98.7% and sensitivity of 60%. The receiver operating characteristic (ROC) curve for the functional model is presented in Fig. 2A.

The structural stepwise model identified five significant predictors of cognitive change slope: greater thickness of the middle temporal gyrus and medial orbitofrontal gyrus, smaller thickness of the entorhinal cortex and lateral orbitofrontal gyrus and smaller hippocampal volume were associated with greater cognitive decline (see Fig. 1 for a visual representation of the five ROIs). The structural unimodal model was significant, *F*(22 259) = 38.56, *P* < 0.001, with a huge effect size (*d*  *=* 2.941)^84^ and explaining 60.5% of the variance in cognitive change. The AIC reflected good quality of fit and moderate complexity (AIC: 552.8). In terms of identifying decliners versus stable individuals, the structural model achieved an odds ratio of 146, *P* < 0.001. With a cut off of 0.773, the model demonstrated an overall classification accuracy of 92.4%, a specificity of 97.3% and a sensitivity of 80%. The ROC curve for the structural model is presented in Fig. 2B.

The neuropsychological stepwise model identified a single significant predictor of cognitive change slope: lower performance on the FCR task was associated with greater cognitive decline. The neuropsychological unimodal model was significant, *F*(10 274) = 53.93, *P* < 0.001, with a very large effect size (*d*  *=* 1.864)^84^ and explained 53% of the variance in cognitive change. The AIC reflected good quality of fit and moderate complexity (AIC: 580.7). For distinguishing decliners from stable individuals, the neuropsychological model had an odds ratio of 1050, *P* < 0.001. With a cut off of 0.933, the model achieved an overall classification accuracy of 98.1%, a specificity of 100% and a sensitivity of 93.3%. The ROC curve for the neuropsychological model is presented in Fig. 2C.

#### Multimodal predictive models

Robust forward stepwise regression analyses of combined modalities were conducted using the predictors identified from the unimodal models (see Table 5). Although sex was included as an interaction variable in the analysis, it did not show a significant effect and was therefore not presented further. The stepwise regression of the functional and structural measures retained five significant predictors of cognitive change slope: higher level of activation in the left hippocampus, lower level of activation in the left inferior frontal gyrus, greater thickness of the middle temporal gyrus, smaller thickness of the lateral orbitofrontal gyrus and a smaller hippocampal volume were associated with greater cognitive decline (see Fig. 1 for a visual representation of the five ROIs). The functional/structural model was significant, *F*(22 234) = 28.37, *P* < 0.001, with a huge effect size (*d*  *=* 2.352)^84^ and explained 55.1% of the variance in cognitive change. The AIC reflected good quality of fit and moderate complexity (AIC: 481.6). The functional/structural model explained significantly more variance than the functional-only model (R^2^ = 52.5% versus 55.1%; *d* = 0.026, *P*  *<* 0.001), but less than the structural-only model (R^2^ = 60.5% versus 55.1%; *d* = 0.054, *P*  *<* 0.001). In terms of identifying decliners versus stable individuals, the functional/structural model had an odds ratio of 511, *P*  *<* 0.001. With a cut off of 0.907, the model achieved an overall classification accuracy of 96.2%, with a specificity of 97.3%, and a sensitivity of 93.3%, which was notably higher than the sensitivity of the functional-only model (60%). The ROC curve for the functional/structural model is presented in Fig. 2D.

**Table 5:** Multimodal predictive models of cognitive change

Models	F test	*P*	R2	AIC	Effect size	OR	ACC (%)	SEN	SPE			
								(%)	(%)	β	T stat	*P*
Functional and structural model	*F*(22, 234) = 28.37	**<0.001**	0.551	481.6	2.352	511	96.2	93.3	97.3			
Activation in the left hippocampus										−0.006	−2.90	**0.005**
Activation in the inferior frontal gyrus										0.006	3.15	**0.002**
Cortical thickness of the middle temporal gyrus										−0.010	−4.32	**<0.001**
Cortical thickness of the lateral orbitofrontal gyrus										0.009	3.83	**<0.001**
Mean volume of the hippocampi										0.004	2.46	**0.016**
Functional and neuropsychological model	F(16, 243) = 38.53	**<0.001**	0.538	478.9	2.318	203.5	91.4	73.3	98.7			
Activation in the left hippocampus										−0.004	−2.31	**0.024**
Activation in the inferior frontal gyrus										0.004	2.16	**0.034**
Sum of free and cued recall scores										0.005	3.27	**0.002**
Structural and neuropsychological model	*F*(19, 256) = 29.35	**<0.001**	0.532	631.3	1.959	300	94.3	80	100			
Cortical thickness of the middle temporal gyrus										−0.008	−3.82	**<0.001**
Cortical thickness of the lateral orbitofrontal gyrus										0.007	3.24	**0.002**
Mean volume of the hippocampi										0.004	3.05	**0.003**
Sum of free and cued recall scores										0.005	3.08	**0.003**
Functional, structural, and neuropsychological model	*F*(25, 225) = 25.42	**<0.001**	0.563	473.3	2.442	243.143	92.4	76.7	98.7			
Activation in the left hippocampus										−0.006	−2.83	**0.006**
Activation in the inferior frontal gyrus										0.006	3.02	**0.004**
Cortical thickness of the middle temporal gyrus										−0.010	−4.75	**< 0.001**
Cortical thickness of the lateral orbitofrontal gyrus										0.010	4.11	**< 0.001**
Mean volume of the hippocampi										0.005	2.68	**0.009**
Sum of free and cued recall scores										0.005	3.04	**0.003**

Bolded values indicate statistically significant results (*P* < 0.05); β (beta) represents the regression coefficient, showing the strength and direction of the relationship between the predictor and the response. The t-stat measures the significance of each coefficient, while *P* indicates the probability of observing such extreme test statistics under the null hypothesis that the coefficient is zero. The *F*-test evaluates the overall significance of the model. R2 indicates the proportion of variance explained by the model. AIC (Akaike’s Information Criterion) measures the model fit, with lower values indicating better fit. Effect size is represented by Cohen’s d. ACC, overall accuracy; OR, odds ratio; SEN, sensitivity; SPE, specificity.

The combination of functional and neuropsychological measures identified three significant predictors of cognitive change: higher activation in the left hippocampus, lower activation in the left inferior frontal gyrus and poorer performance in the FCR task. The functional/neuropsychological model was significant, *F*(16 243) = 38.53, *P* < 0.001, with a huge effect size (*d*  *=* 2.318)^84^ and explained 53.8% of the variance in cognitive change. The AIC reflected good quality of fit and moderate complexity (AIC: 478.9). The functional/neuropsychological model accounted for significantly more variance than the functional-only model (R^2^ = 52.5% versus 53.8%; *d* = 0.013, *P* = 0.033) but did not outperform the neuropsychological-only model (R^2^ = 53% versus 53.8%; *d* = 0.008, *P*  *=* 0.219). In terms of distinguishing between decliners and stable individuals, the odds ratio of the overall model was 203.5, *P*  *<* 0.001. With a cut off of 0.72, the functional/neuropsychological model achieved an overall classification accuracy of 91.4%, with a specificity of 98.7%, and a sensitivity of 73.3%, which was higher than the sensitivity of the functional-only model (60%). The ROC curve for the functional/neuropsychological model is presented in Fig. 2E.

The combination of structural and neuropsychological measures identified four significant predictors of cognitive decline: larger thickness of the middle temporal gyrus, smaller thickness of the lateral orbitofrontal gyrus, smaller volume of the hippocampus and a lower performance on the FCR task. The structural/neuropsychological model was significant, *F*(19 256) = 29.35, *P* < 0.001, with a very large effect size (*d*  *=* 1.959)^84^ and explained 53.2% of the variance in cognitive decline. The AIC reflected good quality of fit and moderate complexity (AIC: 631.3). The structural/neuropsychological model did not outperform the neuropsychological-only model (R^2^ = 53% versus 53.2%; *d* = 0.002, *P*  *=* 0.682), but explained less variance than the structural-only model (R^2^ = 60.5% versus 53.2%; *d* = 0.073, *P*  *<* 0.001). In distinguishing between decliners and stable individuals, the odds ratio for the overall model was 300, *P*  *<* 0.001. With a cut-off of 0.8, the structural/neuropsychological model achieved an overall classification accuracy of 94.3%, with 100% specificity and 80% sensitivity. The ROC curve for the structural/neuropsychological model is presented in Fig. 2F.

Finally, combined analysis of functional, structural and neuropsychological measures identified six significant predictors of cognitive decline: higher activation in the left hippocampus, decreased activation in the left inferior frontal gyrus, larger thickness of the middle temporal gyrus, smaller thickness of the lateral orbitofrontal gyrus, smaller hippocampal volume and lower performance on the FCR task. The multimodal model was significant, *F*(25 225) = 25.42, *P* < 0.001, with a huge effect size (*d*  *=* 2.442)^84^ and explained 56.3% of the variance in cognitive change. The AIC reflected good quality of fit and moderate complexity of the model (AIC: 473.3). The functional/structural/neuropsychological model explained significantly more variance than the functional-only model (R^2^=52.5% versus 56.3%; *d* = 0.038, *P*  *<* 0.001), the neuropsychological-only model (R^2^=53% versus 56.3%; *d* = 0.033, *P*  *<* 0.001), the structural/neuropsychological model (R^2^=53.2% versus 56.3%; *d* = 0.031, *P*  *<* 0.001) and the functional/neuropsychological model R^2^=53.8% versus 56.3%; *d* = 0.025, *P* < 0.001). However, the functional/structural/neuropsychological model did not explain more variance than the functional/structural model (R^2^=55.1% versus 56.3; *d* = 0.012, *P* = 0.064) and explained less variance than the structural-only model (R^2^=60.5% versus 56.3%; *d* = 0.042, *P* < 0.001).

The functional/structural/neuropsychological model effectively identified decliners versus stable individuals, with an odds ratio of 243.143, *P* < 0.001. Using a cut off of 0.753, the model achieved an overall classification accuracy of 92.4%. The model’s specificity was 98.7%, surpassing that of both the structural-only model (97.3%) and the functional/structural model (97.3). Sensitivity was 76.7%, which was higher than the sensitivity of the functional-only model (60%) and the functional/neuropsychological model (73.3%). The ROC curve for the structural/structural/neuropsychological model is presented in Fig. 2G.

## Discussion

This study investigates the role of task-related activation in predicting cognitive decline in individuals with SCD or MCI. Additionally, the study sought to assess and compare the predictive accuracy, sensitivity and predictivity of functional activation with those of brain structure and neuropsychological performance. The study also measured the added value of multimodal models that combine activation with structural and neuropsychological measures.

Our findings indicate that functional activation is a strong predictor of cognitive decline. The predictive accuracy of functional measures was found to be comparable to that of the more commonly used structural and neuropsychological measures when using slope as a continuous measure of decline. However, consistent with our hypothesis, most multimodal models showed greater explained variance than the unimodal functional model. This suggests that unimodal models may not capture all relevant processes, leading to a lower predictive power compared with multimodal models, which integrate diverse markers that reflect different underlying mechanisms or disease effects.

To assess odds ratio, specificity and sensitivity, we used a dichotomous approach, classifying individuals as decliners if they exhibited a clinically significant decline. The neuropsychological model demonstrated the highest odds ratio, likely due to overlap between the cognitive test used to measure decline. Another explanation may be that neuropsychological measures are more predictive of the occurrence of a clinically significant change or imminent progression to dementia. In contrast, functional and structural markers may capture earlier declines that precede clinically significant changes. The same reason could account for the finding of higher explained variance in continuous outcomes but a lower odds ratio with clinically significant decline when comparing the structural model to the functional/structural/neuropsychological model.

When comparing specificity and sensitivity across models, the functional unimodal model demonstrated excellent overall classification with very high specificity but relatively low sensitivity. This indicates that the presence of a functional activation abnormality in an individual almost always signals a future decline, but its absence does not guarantee that decline will not occur. This pattern is often seen in diseases driven by multiple mechanistic pathways, where certain variables are highly indicative of the disease, but are neither the sole nor necessary condition for its development. For example, a mutation in the amyloid precursor protein gene almost inevitably leads to Alzheimer's disease, yet it is rare, and many Alzheimer's disease patients do not carry this mutation.

From a mechanistic perspective, this finding is interesting as it suggests that functional activation may be driven by different underlying mechanisms compared with other predictors. Hyperactivation, for instance, has been proposed to reflect early compensatory processes^6,10,11,18,85^ or excitotoxicity—phenomena that contribute to the disease’s pathology in its earliest phase.^15-17,86^ The observed pattern suggests that whatever mechanism hyperactivation represents, it is not universally present in all individuals experiencing cognitive decline. This could be due to the early and transient nature of hyperactivation,^7,8,10,11,18,64,87^ meaning some participants may have already progressed beyond the stage where hyperactivation occurs.

Due to its high specificity, functional activation, when combined with other markers, could be valuable for clinicians in identifying individuals at higher risk of cognitive decline. This approach might also benefit researchers aiming to identify patients with a strong likelihood of progression for instance, to enrich patient inclusion criteria in pharmacological trials.

This study has several strengths. Notably, it is the first to assess the predictive accuracy of task-related activation in a longitudinal cohort of at-risk individuals. This was made possible by the unique features of the CIMA-Q cohort, one of the few cohorts to include task-related activation data. Another strength lies in the study’s precise comparison of unimodal and multimodal models.

This study also has some limitations. First, the CIMA-Q cohort did not include tau and amyloid brain imaging, which means we cannot confirm whether the cognitive symptoms observed in participants with SCD and MCI are due to underlying Alzheimer's disease pathology, nor can we evaluate protein-based models. The sample size is also relatively small, although it is larger than in most previous functional activation studies in prodromal Alzheimer's disease. The applicability of task-related activation measures in clinical settings also presents challenges. Additionally, the age difference between the SCD and MCI groups could be considered a limitation, as age is closely associated with disease progression and may influence the results. However, age was controlled for as a covariate in our analyses to mitigate this potential bias.

In conclusion, this study highlights the potential role of brain activation anomalies in the early detection of future cognitive change. Functional activation proves to be as predictive as other established predictors of progression, such as structural and neuropsychological markers, offering excellent overall accuracy and high specificity, but lower sensitivity. Interestingly, our results suggest that activation differences might reflect different underlying mechanisms. Future research should focus on examining the role of functional activation in larger cohorts, incorporating amyloid and tau markers, and to better understand the sources of interindividual variability in brain function.

## Supplementary Material

fcaf155_Supplementary_Data

## Data Availability

To access data from the Consortium for the Early Identification of Alzheimer’s Disease (CIMA-Q), requestors must meet specific conditions: they must become a member of CIMA-Q, obtain ethical approval from a Quebec committee (Ministry of Health and Social Services or university), submit an application to the CIMA-Q User Access Committee, and sign a data-sharing agreement. For detailed information on data access procedures, please visit the CIMA-Q website: http://www.cima-q.ca/en/data-cima-q/.

## Appendix

**CIMA-Q Consortium**

Pierre Bellec, Sylvie Belleville, Christian Bocti, Frédéric Calon, Howard Chertkow, Louis Collins, Stephen Cunnane, Simon Duchesne, Pierrette Gaudreau, Serge Gauthier, Sébastien S. Hébert, Carol Hudon, Marie-Jeanne Kergoat, Andréa C. LeBlanc, Nicole Leclerc, Naguib Mechawar, Natalie Philips, Jean-Paul Soucy, Thien T. D. Vu, Louis Verret and Juan M. Villalpando.
