# Supplementary material for: Predicting cognitive change using functional, structural, and neuropsychological predictors
Source: Brain Commun. 2025 Apr 18;7(3):fcaf155. doi: 10.1093/braincomms/fcaf155 (PMC12056721; doi:10.1093/braincomms/fcaf155)
Supplement: fcaf155_Supplementary_Data [file fcaf155_supplementary_data.docx]

**Supplementary Materials 1**

| **Sequence - T1w-3D** | | | | |
| --- | --- | --- | --- | --- |
| **Study** | **CDIP – PCID v3.7** | | | |
| **Vendor** | **Philips** | **Philips** | **Siemens** | |
| **Field Strength** | **3.0T** | **3.0T** | **3.0T** | |
| **Model** | **Ingenia** | **Achieva** | **Trio** | |
| **Version** | **R5** | **3.2.1** | **17** | |
| **Sequence Name** | **3D TFE** | **3D TFE** | **3D MP-RAGE** | |
| **Imaging Options** | **Fast (Sense)** | **Fast (Sense)** | **iPat** | |
| **Pulse Timing** |  |  |  |  |
| **TE (ms)** | **shortest (3.3)** | **shortest (3.3)** | **2.98** | |
| **TR (ms)** | **shortest (7.3)** | **shortest (7.3)** | **2300** | |
| **Flip Angle (°)** | **9** | **9** | **9** | |
| **TI (ms)** | **945** | **945** | **900** | |
| **Scan Range** |  |  |  |  |
| **FOV (in-plane) (mm)** | **256 x 248** | **256 x 248** | **256 x 256** | |
| **Slice thickness (mm)** | **1** | **1** | **1** | |
| **Gap between slices (mm)** | **0** | **0** | **0** | |
| **No. Slices** | **180** | **180** | **192** | |
| **Acquisition** |  |  |  |  |
| **Orientation** | **Sagittal** | **Sagittal** | **Sagittal** | |
| **Matrix size** | **256 x 248** | **256 x 248** | **256 x 256** | |
| **Voxel size [L/R x A/P x I/S] (mm)** | **1 x 1 x 1** | **1 x 1 x 1** | **1 x 1 x 1** | |
| **NEX** | **1** | **1** | **1** | |
| **Acceleration factor (Parallel factor*)** | **2** | **2** | **2** | |
| **Fold-Over direction** | **AP** | **AP** | **AP** | |
| **Reconstruction** |  |  |  |  |
| **Matrix size** | **256** | **256** | **256** | |
| **Voxel size [L/R x A/P x I/S] (mm)** | **1 x 1 x 1** | **1 x 1 x 1** | **1 x 1 x 1** | |
| **Other** |  |  |  |  |
| **Fat Suppression** | **None** | **None** | **None** | |
| **Bandwidth** | **228 Hz/px** | **228 Hz/px** | **240 Hz/px** | |
| **Coil Type** |  |  |  |  |
| **Head** | **x** | **x** | **x** | |
| **Channel** | **15 (Head and Neck)** | **8** | **12** | |
| **Timing** | | | | |
| **Prescan Time+** | **00:30** | **00:30** | **00:30** | |
| **Scan Time** | **06:20** | **06:17** | **05:21** | |
| **Total Time (min)** | **06:50** | **06:47** | **05:51** | |

| **Sequence - Task fMRI** | | | | | | | | | |
| --- | --- | --- | --- | --- | --- | --- | --- | --- | --- |
| **Study** | **CIMA-Q Add-on** | | | | | | | | |
| **Vendor** | **Philips** | **Philips** | **Philips** | **Philips** | **Philips** | **Philips** | **Siemens** | **Siemens** | **Siemens** |
| **Field Strength** | **3.0T** | **3.0T** | **3.0T** | **3.0T** | **3.0T** | **3.0T** | **3.0T** | **3.0T** | **3.0T** |
| **Model** | **Ingenia** | **Ingenia** | **Ingenia** | **Achieva** | **Achieva** | **Achieva** | **Trio** | **Trio** | **Trio** |
| **Version** | **R5** | **R5** | **R5** | **3.2.3** | **3.2.3** | **3.2.3** | **17** | **17** | **17** |
| **Sequence Name** | **FFE EPI** | **FFE EPI** | **B0 Map** | **FFE EPI** | **FFE EPI** | **B0 Map** | **fMRI EPI** | **fMRI EPI** | **GRE field mapping** |
| **Imaging Options** |  |  |  |  |  |  |  |  |  |
| **Pulse Timing** | | | | | | | | | |
| **TE (ms)** | **25** | **25** | **4.6** | **25** | **25** | **4.6** | **25** | **25** | **4.92 / 7.38** |
| **TR (ms)** | **2500** | **2500** | **475** | **2500** | **2500** | **475** | **2500** | **2500** | **476** |
| **Flip Angle (°)** | **90** | **90** | **60** | **90** | **90** | **60** | **90** | **90** | **60** |
| **TI (ms)** | **-** | **-** | **-** | **-** | **-** | **-** | **-** | **-** | **-** |
| **Scan Range** |  |  |  |  |  |  |  |  |  |
| **FOV (in-plane) (mm)** | **240 x 240** | **240 x 240** | **240 x 240** | **240 x 240** | **240 x 240** | **240 x 240** | **222 x 222** | **222 x 222** | **222 x 222** |
| **Slice thickness (mm)** | **3** | **3** | **3** | **3** | **3** | **3** | **3** | **3** | **3** |
| **Gap between slices (mm)** | **0.3** | **0.3** | **0.3** | **0.3** | **0.3** | **0.3** | **0** | **0** | **0** |
| **No. Slices** | **41** | **41** | **41** | **41** | **41** | **41** | **41** | **41** | **45** |
| **Acquisition** |  |  |  |  |  |  |  |  |  |
| **Orientation** | **AC-PA minus 20°** | **AC-PA minus 20°** | **AC-PA minus 20°** | **AC-PA minus 20°** | **AC-PA minus 20°** | **AC-PA minus 20°** | **AC-PA minus 20°** | **AC-PA minus 20°** | **AC-PA minus 20°** |
| **Matrix size** | **80 x 80** | **80 x 80** | **80 x 80** | **80x79** | **80x79** | **80 x 80** | **74** | **74** | **74** |
| **Voxel size [L/R x A/P x I/S] (mm)** | **3 x 3 x 3** | **3 x 3 x 3** | **3 x 3 x 3** | **3 x 3 x 3** | **3 x 3 x 3** | **3 x 3 x 3** | **3 x 3 x 3** | **3 x 3 x 3** | **3 x 3 x 3** |
| **NEX** | **1** | **1** | **1** | **1** | **1** | **1** | **1** | **1** | **1** |
| **Acceleration factor (Parallel factor*)** | **1.8** | **1.8** | **1** | **1.3** | **1.3** | **1** | **1** | **1** | **1** |
| **Fold-Over direction** | **AP** | **First AP / Second PA** | **AP** | **AP** | **First AP / Second PA** | **AP** | **AP** | **First AP / Second PA** | **AP** |
| **Reconstruction** |  |  |  |  |  |  |  |  |  |
| **Matrix size** | **80** | **80** | **80** | **64** | **64** | **80** | **74** | **74** | **74** |
| **Voxel size [L/R x A/P x I/S] (mm)** | **3 x 3 x 3** | **3 x 3 x 3** | **3 x 3 x 3** | **3 x 3 x 3** | **3 x 3 x 3** | **3 x 3 x 3** | **3 x 3 x 3** | **3 x 3 x 3** | **3 x 3 x 3** |
| **Other** |  |  |  |  |  |  |  |  |  |
| **Fat Suppression** | **Fat Sat. SPIR** | **Fat Sat. SPIR** | **None** | **Fat Sat. SPIR** | **Fat Sat. SPIR** | **None** | **Fat Sat.** | **Fat Sat.** | **None** |
| **Bandwidth (Hz/Px)** | **39.1** | **39.1** | **294.8** | **25.2** | **25.2** | **294.8** | **2502** | **2502** | **268** |
| **Number of acquisitions** | **300** | **4** | **-** | **300** | **4** | **-** | **310** | **4** | **-** |
| **Coil Type** |  |  |  |  |  |  |  |  |  |
| **Head** | **x** | **x** | **x** | **x** | **x** | **x** | **x** | **x** | **x** |
| **Channel** | **15 (Head and Neck)** | **15 (Head and Neck)** | **15 (Head and Neck)** | **8** | **8** | **8** | **12** | **12** | **12** |
| **Timing** | | | | | | | | | |
| **Prescan Time+** | **00:30** | **00:30** | **00:30** | **00:30** | **00:30** | **00:30** | **00:30** | **00:30** | **00:30** |
| **Scan Time** | **12:35** | **00:17** | **01:17** | **12:35** | **00:17** | **01:17** | **13:03** | **00:17** | **01:17** |
| **Total Time (min)** | **13:05** | **00:47** | **01:47** | **13:05** | **00:47** | **01:47** | **13:33** | **00:47** | **01:47** |
| **Comments:** | **Tested 22-AVR-15** | **Tested 22-AVR-15** | **Tested 22-AVR-15** | **Tested 10-AVR-15** | **Tested 10-AVR-15** | **-** | **Tested 10-MAR-15** | **Tested 10-MAR-15** | **Tested 10-MAR-15** |
|  |  |  |  |  |  |  |  |  |  |

| **Sequence - 2D FLAIR** | | | | |
| --- | --- | --- | --- | --- |
| **Study** | **CDIP – PCID v3.7** | | | |
| **Vendor** | **Philips** | **Philips** | **Siemens** | |
| **Field Strength** | **3.0T** | **3.0T** | **3.0T** | |
| **Model** | **Ingenia** | **Achieva** | **Trio** | |
| **Version** | **R5** | **3.2.3** | **17** | |
| **Sequence Name** | **2D FLAIR** | **2D FLAIR** | **2D TDF** | |
| **Imaging Options** | **Fast (Sense)** | **Fast (Sense)** | **iPat** | |
| **Pulse Timing** |  |  | |  |
| **TE (ms)** | **125** | **125** | **123** | |
| **TR (ms)** | **9000** | **9000** | **9000** | |
| **Flip Angle (°)** | **150** | **150** | **165** | |
| **TI (ms)** | **2500** | **2500** | **2500** | |
| **Scan Range** |  |  | |  |
| **FOV (in-plane) (mm)** | **240 x 210** | **240 x 210** | **240 x 240** | |
| **Slice thickness (mm)** | **3** | **3** | **3** | |
| **Gap between slices (mm)** | **0** | **0** | **0** | |
| **No. Slices** | **48** | **48** | **48** | |
| **Acquisition** |  |  | |  |
| **Orientation** | **Oblique axial** | **Oblique axial** | **Oblique axial** | |
| **Matrix size** | **256x224** | **256x222** | **256 x 256** | |
| **Voxel size [L/R x A/P x I/S] (mm)** | **0.94 x 0.95 x 3** | **0.94 x 0.95 x 3** | **0.94 x 0.94 x 3** | |
| **NEX** | **1** | **1** | **1** | |
| **Acceleration factor (Parallel factor*)** | **2** | **2** | **2** | |
| **Fold-Over direction** | **RL** | **RL** | **RL** | |
| **Reconstruction** |  |  | |  |
| **Matrix size** | **256** | **256** | **256** | |
| **Voxel size [L/R x A/P x I/S] (mm)** | **0.94 x 0.94 x 3** | **0.94 x 0.94 x 3** | **0.94 x 0.94 x 3** | |
| **Other** |  |  | |  |
| **Fat Suppression** | **None** | **None** | **None** | |
| **Bandwidth** | **164 Hz/px** | **242 Hz/px** | **222 Hz/px** | |
| **Coil Type** |  |  | |  |
| **Head** | **x** | **x** | **x** | |
| **Channel** | **15 (Head and Neck)** | **8** | **12** | |
| **Timing** | | | | |
| **Prescan Time+** | **00:30** | **00:30** | **00:30** | |
| **Scan Time** | **04:48** | **04:12** | **04:05** | |
| **Total Time (min)** | **05:18** | **04:42** | **04:35** | |
| **Comments:** | **Set parameter "image filter" at Weak** |  |  | |
|  |  |  |  |  |
